# Healthcare staff perceptions of the hospital food environment: a narrative systematic review

**DOI:** 10.1017/S136898002500031X

**Published:** 2025-03-20

**Authors:** Lorraine McSweeney, Marta Buczkowska, Laura Denning, Millie Elcock, Suzanne Spence

**Affiliations:** 1 Human Nutrition and Exercise Research Centre, Population Health Sciences, Newcastle University, Newcastle upon Tyne, UK; 2 School of Biomedical, Nutrition and Sports Sciences, Newcastle University, Newcastle upon Tyne, UK

**Keywords:** Hospital staff, Hospital food environment, Perceptions, Health and well-being

## Abstract

**Objective:**

To understand healthcare staff perspectives of their hospital food environment and the impact of these perceptions on their food choice, health and well-being.

**Design:**

A narrative systematic review.

**Setting:**

Publications were eligible for inclusion if participants were hospital-based staff, and all job roles were eligible, including both clinical and non-clinical staff. Both public and private hospitals in the UK, the USA or Australia were included.

**Participants:**

Clinical and non-clinical staff employed in hospitals.

**Results:**

A systematic search was carried out across four databases: OVID Medline, CINAHL, PsycInfo and Scopus. Grey literature screening was completed via Google and Google Scholar. Eleven studies were included and were predominantly from the UK. Setting sizes varied or were unknown, and participant numbers varied (*n* 16 to *n* 1158) or were unknown. Most participants were nurses. Methods included reports, surveys, focus groups and interviews. The main themes identified were accessibility to food, diversity of food choices, free foods used to boost staff morale and job role influencing engagement with the food environment. Staff reported issues around canteen opening hours, limited healthy food options and free food on wards, causing extra calories to be consumed. Irregular breaks and staffing shortages affected hospital staff’s ability to engage with the wider food environment, resulting in reliance on convenience foods and snacks.

**Conclusions:**

The current hospital food environment does not facilitate healthy dietary practices and is perceived by staff as a barrier to healthy eating. The hospital food environment requires adaptation to reflect a 24-hour workplace.

Global prevalence of overweight and obesity has nearly tripled since 1975^(1)^. Currently, more than 1·9 billion people, or 39 % of the global population, are considered overweight or obese^(2)^. The prevalence of obesity is higher in high-income countries. In 2018, 67 % of the Australian adult population were living with overweight or obesity^(3)^, compared with 73 % of Americans^(4)^ and 63 % of Britons^(5)^. Healthcare staff are not exempt from this trend, as statistics show that nearly one in four nurses both in the UK and the USA are living with obesity^(6,7)^. Moreover, a study conducted in Australia, New Zealand and the UK found that nurses and midwives are more likely to be living with overweight or obesity than the general public^(8)^. Staff health and well-being is a priority, with staffing shortages a concern in the healthcare sector along with an ageing workforce^(9)^. Therefore, prioritising the health of hospital staff is key to securing a future workforce to deliver quality care to patients^(10)^.

The staff hospital food environment is a growing concern in relation to their health and well-being. The hospital food environment includes where food and drinks are purchased, such as restaurants, on-site shops and vending machines, alongside facilities where staff can prepare and consume their own food^(11)^. It also refers to the availability, advertising and cost of products^(12)^. In 2018, 39 % of National Health Service (NHS) staff surveyed, stated the food and catering facilities in their hospital were poor^(11)^. Workplace environment is a determinant of health as described by the Dahlgren and Whitehead model^(13)^, and therefore, it can be targeted to improve the health and well-being of employees and subsequent improvements may reduce health inequalities as hospitals have a diverse workforce^(14)^.

In the UK, NHS England hospital food standards have been in place since 2014^(15)^, addressing the quality of food for patients, staff and visitors. However, the emphasis when created was directed at patients, with improvements measured by patient-led assessments only^(15)^. In 2017, this shifted to include staff health and well-being indicators^(16,17)^, suggesting increased awareness of staff requirements. An independent review of NHS hospitals was published in 2020^(11)^, reporting the continuing challenges within hospitals to cater for the diverse needs of patients, staff and visitors. The report indicated that night shift staff may be the least catered for, having no access to hot food and options being limited to vending machines^(11)^. In 2022, eight new food standards were published by NHS England, including 24/7 access to hot and cold food for staff^(18)^. However, none of the standards seek to gain feedback from the staff. These standards were published after the campaign #NoHungryNHSStaff^(19)^, led by NHS staff campaigning for improved availability and affordability of healthy foods, particularly across night shifts and weekends^(19)^, highlighting staff’s continuing needs. NHS England has previously suggested that improving the hospital food environment would support staff to make healthier choices^(17)^. Research has shown that changes in cost, availability and accessibility of healthy food choices have a positive influence on purchasing trends of staff^(20)^. Furthermore, subsidised healthier food choices have been suggested to impact staff morale, well-being and absence rates^(18)^; however, evidence is limited. Research exploring the impact of the hospital food environment on hospital staff’s dietary behaviour has increased in recent years, with a growing recognition of the importance of the relationship between the workplace food environment and employees’ productivity and well-being^(9,21–23)^. However, there are currently no systematic reviews that examine hospital staff’s views regarding the food available to them at work and the impact it may have on their mental and physical health. Therefore, this systematic review aims to explore (1) hospital staff’s perceptions of the hospital food environment and (2) the impact it may have on their health and well-being.

## Methods

A protocol was developed following the ‘Preferred Reporting Items for Systematic Reviews and Meta Analyses’ (PRISMA) framework^(24)^. The protocol was registered on Prospero on 27 February 2023. Registration number CRD42023400550.

### Eligibility criteria

Eligibility criteria followed the Participant, Intervention, Context (PICo) structure^(25)^. Publications were eligible for inclusion if participants were hospital-based staff, and all job roles were eligible, including both clinical and non-clinical staff. Both public and private hospitals were included. Publications exploring staff perceptions of their hospital food environment were included, and studies that solely investigated staff intake were excluded. The following were also excluded: staff working in community settings and perceptions of patient food provision. Intervention studies were excluded, as the review focus was the hospital food environment. Qualitative and mixed methods studies were eligible for inclusion. To reflect the current environment, only studies from 2010 onwards were included. Studies were restricted to the UK, USA and Australia, as they are all English-speaking countries with high obesity prevalence. Therefore, only studies written in the English language were included.

### Search strategy

A systematic search was carried out across four databases: OVID Medline, CINAHL, PsycInfo and Scopus. The search strategy included keywords from the following concepts: occupation, perception, food environment and setting. The full search strategy can be found in Appendix 1. In addition, grey literature screening was completed via Google and Google Scholar on 09 February 2023, using our systematic review title as the search term. Only the first five pages of results were screened, due to time constraints.

### Screening methodology

**Figure 1:**
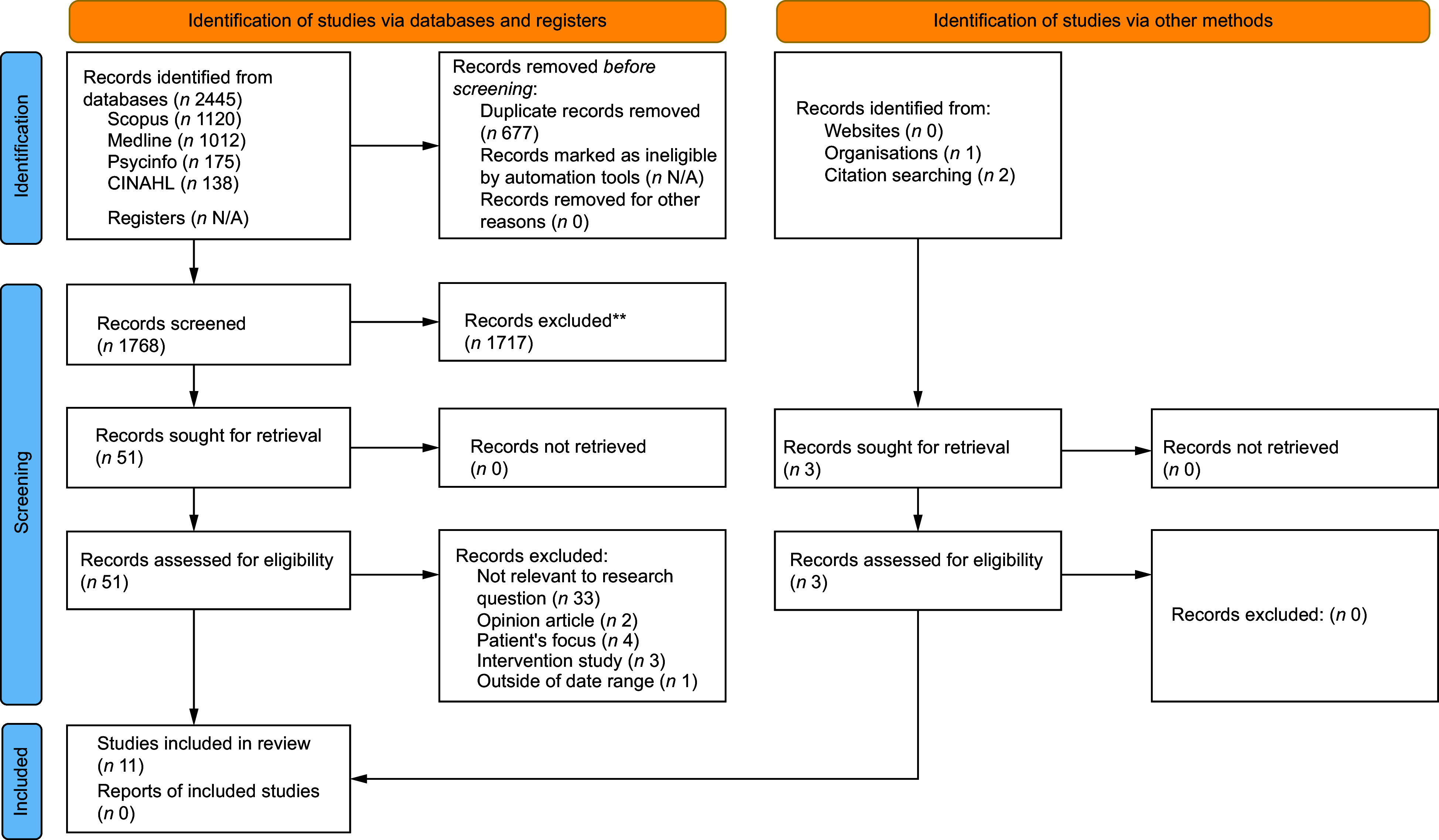
PRISMA flowchart recording the screening process.

Publications identified from the search were exported to Endnote 20^(26)^. Screening via title and abstract against the eligibility criteria was conducted by three researchers (MB, LD, ME). Eligible studies underwent full-text screening. Two researchers screened each text independently; discrepancies were discussed between researchers (MB, LD, ME). Reference lists of eligible texts, after full-text screening, were screened to search for additional papers fit for inclusion (MB, LD, ME). Two studies were identified in this way. The screening process was documented in a PRISMA flowchart^(23)^ (Figure 1).

### Data extraction

Data from eligible studies was extracted using a standardised template on Microsoft Excel^(27)^. Extracted data included citations, study aims, methodology, participant demographics, outcomes and main findings. Data were extracted from each publication twice by two researchers independently, and discrepancies were discussed in a meeting between researchers.

### Risk of bias

Risk of bias was completed simultaneously with data extraction using the ‘Quality Checklist for Primary Research’^(28)^. The tool assessed the following domains: relevance and validity, including the relevance of the topic to the dietetic field and methodology used. Completion of the tool resulted in a score to define the risk of bias as high, neutral or low. Additionally, the quality of studies was assessed using the Critical Appraisal Skills Programme tool for qualitative research^(29)^. The tool assessed the validity of results and the research value, aiding the completion of the risk of bias assessment. The tools were completed by two researchers independently, and the results were discussed between all three researchers to resolve discrepancies. Studies were not excluded based on the risk of bias.

### Data analysis and synthesis

This systematic review synthesised qualitative data from focus groups, interviews, questionnaires, surveys and online comments. Due to the qualitative nature of this review and the heterogeneity of designs and participants in the included studies, data were analysed and synthesised using thematic analysis. It is a well-established approach to synthesising qualitative data, widely used in research investigating people’s perceptions. The analysis was performed following the framework developed by Braun and Clarke in 2006^(30)^. The articles were coded for the presence of reoccurring hospital staff’s perceptions regarding their workplace food environment (e.g. cost, accessibility) and its influence on their health and well-being (e.g. weight gain, stress). Subsequently, the identified codes were grouped into descriptive themes, then refined into five analytical themes and an additional five sub-themes to capture the essence of the data.

## Results

The search generated 2445 publications, of which 677 were duplicates. After screening the titles and abstracts of 1717 articles, fifty-one publications were identified for full-text screening. Forty-three articles were excluded in the process, predominantly due to being irrelevant to the research question. Eight publications^(20,31–37)^ met the inclusion criteria. An additional two papers^(38,39)^ were found following the screening of the references of eligible articles, and one publication was identified through the grey literature scoping search^(11)^.

### Participant characteristics

Key study characteristics were collated (Table 1). The review consists of a total number of 2244 participants from eight studies^(20,31,33,34,36–39)^. The study populations comprised nurses, doctors, support staff, allied health workers and non-clinical employees. Additionally, the review includes twenty-one comments on Talk Health and Care platform,^(11)^ 314 votes in a Twitter poll,^(11)^ fourteen online comments^(11)^ and an unclear number of the Nursing Standard readers^(32,35)^ Studies were conducted in acute (*n* 1)^(31)^ community (*n* 1)^(34)^ and teaching hospitals (*n* 2)^(38,39)^. Seven publications did not explicitly report the type of setting^(20,32,34,35,37–39)^.

**Table 1:** Characteristics of included studies

Citation, Country	Site	Study design/ type of publication	Data collection method	Study aim	Participants	Key perceptions	Risk of bias analysis
Cubitt *et al.* (2021), UK^(31)^	Acute NHS Trust	Cross-sectional	Survey	Identify factors influencing doctors’ well-being during COVID-19	Doctors, *n* 242	Staff considered free food available at the workplace as morale boosting.	Low
Dean (2014), UK^(32)^	N/A	Magazine article	Unclear	N/A	*‘Nursing Standard’* readers, number not reported	The hospital food environment is perceived as unhealthy, and staff acknowledged numerous barriers to healthy eating. Those included limited availability of healthy options, poor quality and taste of the food offered, shift work, high price of healthy foods and time constraints.	High
Department of Health and Social Care (2020), UK^(11)^	N/A	Report	Talk Health and Care platform, Twitter poll	To offer hospital staff the opportunity to share their experience of food provision for staff in hospitals.	Twenty-one comments on Talk Health and Care platform, 314 votes on Twitter polls and fourteen additional comments	The hospital food environment was perceived negatively by the staff. They reported limited access to the cafeteria during nights and weekends, lack of consideration of staff’s dietary requirements and scarcity of healthy choices. Staff also reported excessive cost and poor quality of food, not having access to proper food facilities and being too busy to eat at work. Some staff praised their hospitals for the healthy food options on offer and community gardens.	High
Horton Dias & Dawson (2020), USA^(33)^	Seven hospitals in South Carolina	Qualitative, descriptive study	Individual interviews and focus groups	To explore hospital shift nurses’ experiences with healthy eating while at work and nurses’ perceived dietary influencers in the hospital setting.	Nurses, *n* 21	Nurses reported numerous problems associated with the hospital food environment – namely limited access to cafeteria during shifts, low food quality, unhealthy snacks available in vending machines, lack of access to high-quality options during night shifts, high cost of food, lack of time to consume meals, free food used as currency by the management and unlimited access to unhealthy free foods at wards. Nurses also described using food to cope with stress and tiredness at work.	Neutral
Jordan *et al.* (2016), USA^(34)^	Midwestern United States community hospital	Cross-sectional	Survey	To assess nurses’ health status, health behaviour, self-reported stress levels, coping techniques, perceived coping effectiveness and situation-specific self-efficacy to cope with workplace-related stress.	Nurses, *n* 120	The hospital food environment was considered unsatisfactory due to lack of access to the cafeteria during shifts, poor food quality, limited availability of healthy options, high food costs or lack of time to consume meals. Eating was a common stress-coping strategy.	Neutral
Keogh (2014), UK^(35)^	N/A	Magazine article	Online comments	N/A	*‘Nursing Standard’* readers, number not reported	The hospital food environment was described as abundant in accessible healthy options by one reader. Readers also reported a lack of breaks due to staff shortages, which was associated with increased consumption of sugary snacks for energy boosts.	High
Mittal *et al.* (2018), UK^(36)^	Four NHS hospitals within the London area	Cross-sectional	Survey	(1) To assess the status of cardiovascular risk factors in NHS staff, measure their compliance with national dietary and physical activity guidelines and perform a comparison between clinical and non-clinical staff with respect to these parameters. (2) To assess the personal and organisational factors that the staff perceives to be barriers to a healthy lifestyle.	Doctors and nurses (51 %) and non-clinical staff (49 %), *n* 1158	Limited availability of healthy food options in the canteen and lack of managerial support were perceived as the main barriers to healthy eating in the hospital environment.	Neutral
Nahm *et al.* (2012), USA^(38)^	Community-based urban teaching hospital	Cross-sectional	Survey	To assess nurses’ selected self-care behaviours, focusing on diet, exercise, stress and weight and their preferred strategies to manage those behaviours.	Nurses, *n* 169	Nurses described numerous barriers to healthy eating at work. These involved a lack of time for regular meals during shifts, high prices of fresh food and lack of access to cafeteria during night shifts. Eating was reported as one of the main stress-coping techniques.	Neutral
Power *et al.* (2017), UK^(39)^	Aberdeen Royal Infirmary teaching hospital	Semi-structured qualitative review	Interviews	To systematically explore the most salient determinants of unhealthy eating and physical activity behaviour in hospital-based nurses.	Nurses, *n* 16	Nurses identified key determinants of unhealthy eating present within the hospital food environment. These included considerable distance to healthy food options, expense of the canteen, limited availability of healthy foods, unhealthy foods offered by colleagues, patients and their families and lack of time to consume food at work, which resulted in overeating at the end of shifts.	Low
Torquati *et al.* (2016), Australia^(37)^	Three hospitals in the Brisbane Metropolitan Area	Qualitative	Focus groups	To gain an understanding of nurses’ determinants contributing to unhealthy diet and being insufficiently active and use the data to inform a needs assessment for a future workplace health promotion programme.	Nurses, *n* 17	Key barriers to healthy eating identified by nurses included lack of time to take breaks and to eat healthily during shifts, access to high-energy snacks on wards, lack of social support and unsupportive workplace culture. Workers also reported using food to cope with negative emotions and to keep awake during night shifts.	Neutral
Utter *et al.* (2022), Australia^(20)^	Mater South Brisbane hospital	Secondary analysis of a health and well-being survey	Survey	To understand more about the significance of the hospital food environment to the nutritional well-being of staff and explore opportunities for improvement.	Nurses (39 %), hospital support staff (24 %), allied health workers (17 %), professional support staff (15 %) and doctors (10 %), *n* 501	The hospital food environment was perceived as unsupportive of healthy eating habits. Staff discussed high food cost, no access to out-of-hours options, too short lunch breaks, lack of fresh options and limited availability of food facilities. Staff perceived potential initiatives to improve hospital food environment useful.	Neutral

### Countries

Six publications^(11,31,32,35,36,39)^ were based in the UK. Three studies were conducted in the USA^(33,34,38)^ and two in Australia^(20,37)^.

### Study design and data collection methods

The study designs comprised four cross-sectional studies^(31,34,36,38)^, three qualitative studies^(33,37,39)^ and one secondary analysis of a health and well-being survey^(20)^. Additionally, this review includes two magazine articles^(32,35)^ published in Nursing Standard and one report produced by the Department of Health and Social Care^(11)^. The survey was the primary data collection method. (*n* 5)^(20,31,34,36,38)^. Other methods included interviews (*n* 1),^(39)^ focus groups (*n* 1)^(37)^ and interviews and focus groups combined (*n* 1)^(33)^. Staff opinions were also collected from Twitter and extracted from online comments (*n* 1)^(11)^. Two publications did not report the data collection methods^(32,35)^. However, upon approaching via LinkedIn on 21·February·2023, the author of one article^(35)^ stated that the comments were possibly extracted from the Nursing Standard’s social media accounts.

### Quality appraisal

According to the Quality Criteria Checklist, Primary Research^(40)^ and the Critical Appraisal Skills Programme Qualitative Checklist,^(29)^ two publications were classified as having low risk of bias,^(31,39)^ six were considered neutral,^(20,33,34,36–38)^ whilst three were ranked as having a high risk of bias^(11,32,35)^. The main types of bias included selection (*n* 8),^(11,20,32,33,35–38)^ response (*n* 6)^(20,33,34,37,38)^ and reporting (*n* 2)^(32,35)^ bias. The perceptions included in the publications with a high risk of bias^(11,32,35)^ cannot be verified as coming from hospital staff. Five publications ranked as having neutral risk of bias scores^(20,33,34,37,38)^ relied on a subjective definition of ‘healthy eating’. Additionally, three studies reported low response rates^(20,36,38)^.

### Thematic analyses

Five main themes and five sub-themes (Table 2) were identified.

**Table 2:** Main themes and sub-themes identified from data analysis

Main themes	Sub-themes
Lack of affordable options	
Food accessibility	Limited access to cafeteria
	Access to staff facilities
	Time constraints
Availability of nutritious *v*. non-nutritious foods	
Expressing gratitude with food	
Eating as a coping mechanism	Emotional eating
	Energy-boosting snacks

### Lack of affordable options

Hospital staff perceived the food provision from hospital canteens as expensive^(20,32,34,38,39)^ or ‘totally overpriced’^(20)^. It was often highlighted that healthy and fresh foods usually cost more than less nutritious options^(32,34,38,39)^. It was also reported that perceptions of the cost influenced the hospital staff’s dietary habits, as nurses highlighted that the high price of food prevented them from eating healthily^(34,39)^. Moreover, the study by Utter *et al.* ^(20)^ described how introducing more affordable meals was supported by the large majority of staff.

### Food accessibility

#### Limited access to cafeteria

Hospital workers consistently reported limited access to cafeterias and diners as a common problem they faced in the workplace^(11,31–34,38)^. The main difficulty experienced by the staff was limited opening hours.

*‘Cafeteria is only open very limited time. I come here at 2pm some days and it is closed…’* ^(33)^.

This was perceived as challenging especially by the nurses, often working night shifts^(11,31,33,38)^. Limited access to the cafeteria was also perceived as a barrier to consuming regular meals^(38)^ and as a driver towards buying unhealthy vending options available around the clock^(32,33)^.

#### Access to staff’s facilities

Hospital staff described the lack of access to the staff kitchens,^(11)^ and self-catering facilities, such as microwaves or fridges and food storage equipment^(41)^, as an important barrier to eating healthily at work^(11)^. Moreover, British doctors highlighted that limited access to regularly disinfected staff canteens significantly influenced their well-being during the COVID-19 pandemic^(31)^.

Workers suggested that providing access to hot water, milk or coffee would significantly improve the hospital food environment^(41)^. Additionally, participants supported creating outdoor eating areas and on-site fruit and vegetable gardens. Similar initiatives already in place appeared to be positively received in the Twitter poll, where commenters praised their hospitals for organising gardening projects^(11)^.

#### Time constraints

Employees frequently reported being *‘too busy’* to take breaks to eat^(33,35,37–39)^ or working entire shifts without eating^(33,39)^. Nurses described that their care duties impacted on their ability to take breaks and that their limited lunch breaks were often interrupted^(33)^. Additionally, nurses reported spending a significant part of their breaks getting to and from the cafés located at a considerable distance from the wards^(33,41)^.

Insufficient breaks^(11,34)^ and lack of set mealtimes were considered important barriers to healthy eating at hospitals^(37–39)^. Nurses highlighted that after hours of not eating at work, they overate at night, which reportedly led to low sleep quality, gastric reflux and weight gain^(33)^. Moreover, nurses reported choosing unhealthy snacks and eating ‘in panic’ during busy shifts^(33)^.

*‘When you haven’t taken a bathroom break in six hours, it’s hard to (…) pour dressing over the salad and eat it, as opposed to just grabbing a Snicker’s bar’* ^(33)^.

### Availability of nutritious *v*. non-nutritious foods

Staff consistently highlighted dissatisfaction with the taste of available food, with healthy options being described as *‘not very appetizing’* ^(33,34)^. Additionally, limited choice of nutritious foods was considered a barrier to eating healthily at work^(11,34–37)^.

Nurses described how it took too long to find healthy foods^(34,39)^ and the options they could find were considered unattractive^(32,33)^ or non-nutritious^(32)^. On the contrary, in two publications,^(33,35)^ some staff members were satisfied with the quality of healthy options available at their place of work. Employees also expressed concerns about the abundance of unhealthy, calorie-dense choices. Staff were particularly unhappy by the quality of the vending machine options^(32,33)^ and strongly supported improving their healthfulness^(41)^.

*‘Usually your choices in the vending machine are junk.’* ^(33)^.

Moreover, Utter *et al.* ^(20)^ found purchasing food at work to be inversely associated with healthy eating. Improvements suggested were ‘more natural ingredients’ and ‘fresh sandwiches made to your choice.’ Although staff favoured increased healthy options, reducing unhealthy options was less favourable as only 19 % supported decreased unhealthy vending machine options. Bringing food from home was perceived as the best strategy for healthy eating^(33)^. Mittal *et al.* ^(36)^ and Power *et al.* ^(39)^ reported that limited healthy options and the presence of unhealthy foods triggered overconsumption.

Another frequently raised concern was the unhealthy food sometimes brought to work by staff to share with colleagues. Nurses considered it as both an important part of the ward culture and a barrier to healthy eating^(33)^.

*‘You go into work (…) and there’s donuts (…) on the table and you just want one!’* ^(33)^.

### Expressing gratitude with food

Staff highlighted the use of free food to express gratitude – either by the management^(31,33)^ or by the patients and their families^(33,39)^. A British study conducted during the COVID-19 pandemic^(31)^ described that the access to free food and drinks provided by the management made the staff feel rewarded and was considered as *‘morale boosting’.*

*‘Free coffee (…) makes me feel that my contribution is actually respected’* ^(31)^.

Two studies^(33,39)^ reported that patients often gave nurses chocolates and candies as a way of showing gratitude. Staff perceived this phenomenon as an important barrier to healthy eating^(39)^.

### Eating as a coping mechanism

#### Emotional eating

Publications exploring the nurses’ perceptions of the hospital food environment^(33,34,37,38)^ reported that nurses use food to relieve negative emotions experienced at work. While feeling overwhelmed or upset, staff turned to ‘comfort food’, such as sweets or junk food^(33,37)^.

*‘If something is upsetting you at work, you make comfort eat [sic]… You tend to have sugary or salty.’* ^(37)^.

The study by Jordan *et al.* ^(34)^ found that nurses consumed more junk food or simply more food than usual after being exposed to work-related stress. Similarly, Nahm and colleagues^(38)^ reported that eating was an important strategy to cope with stress and other negative emotions.

#### Energy-boosting snacks

Night shift workers in three publications^(33,35,37)^ described using food and drinks as energy boosters. They reported consuming products high in simple carbohydrates, such as crisps or candies, fizzy drinks and caffeinated beverages to help them stay awake and alert throughout the night. Healthcare staff highlighted that turning to ‘high-carb’ options negatively influenced their well-being,^(33)^ or even made them feel *‘revolting’* ^(37)^. Some employees also associated late-night snacking with night shift nurses being more overweight than the day shifters^(33)^.

## Discussion

The findings of this systematic review suggest that hospital staff perceive the hospital food environment as inadequate and a barrier to healthy eating^(31–39,41)^. Healthy food that is financially and physically accessible was desired by staff^(11,33,34,38,39,41)^. Despite this, current healthy food options were reported as limited^(32,33,41)^.

Alongside workplace stress, the hospital food environment was perceived as making healthy eating the more difficult choice, and bringing in healthy homemade options was thought of as the best strategy for healthy eating^(33)^. Earlier research found hospital doctors reported similar issues around canteen opening hours, food variety and lack of breaks as barriers to healthy eating^(42)^. Furthermore, staff perceptions of too many unhealthy options are supported by recent research, which reported an abundance of unhealthy foods in South Carolina hospitals^(43)^. Additionally, comparable issues in terms of accessibility of healthier options due to limited cafeteria opening hours were reported^(43)^. Similarly, in the UK, research has revealed hospital canteen lunches can provide over half of the daily recommended intake of fat and salt^(44)^. This systematic review adds to previous findings on the prevalence of unhealthy foods in hospitals, by showing that staff perceive this as a barrier to healthy eating and are supportive of healthier options.

A recent systematic review of workplace interventions suggests that they can have small, positive effects. However, there is no ‘one-size fits all’ and owing to the unique social and environmental assets of a particular workplace, interventions should be tailored^(45)^. As highlighted in this review, there are a multitude of factors that influence colleague engagement with the hospital food environment, particularly staff’s professional responsibilities which can act as a barrier^(11,32–35,37–39,41)^. Staff reported irregular breaks, work overload and difficulties leaving their units/departments, due to staffing shortages, as barriers to healthy eating. This finding is significant and challenges the effectiveness of improving the hospital food environment if staff are unable to access provision unless wider issues are resolved, or more novel food systems, such as pre-ordering services, are implemented. Our findings suggest that the hospital food environment has not adapted to the current workplace environment to ensure staff are able to access nutritious food regardless of their job pressures. Previous studies identified high workload as a cause for nurses skipping meals, and nurses perceived this as detrimental to their well-being^(46)^. The impact of the COVID-19 pandemic was highlighted by some workers^(31)^ which, when mediated by occupational stressors, was reported to lead to changes in body weight in some workers^(47)^. The additional stress staff faced during the pandemic increased negative interactions with the work food environment, with reports of increased grazing/snacking and fast-food consumption in US workers^(48)^.

Intervention studies are required to simultaneously target workload, protection of break times and staffing initiatives to allow staff to interact with the food environment, to maximise the impact on dietary behaviour. Furthermore, this systematic review highlights the additional challenges of working out of hours that staff face. Night shift staff reported that cafeterias are usually closed at night^(11,34,38,39,41)^, which resulted in reliance on convenience foods such as those in vending machines^(37,43)^. Vending machines are typically stocked with products high in fat, salt and sugar and are non-compliant with nutrition policies^(41,49)^. Collectively, these data suggest night shift staff are disadvantaged in terms of access to healthy options. This finding is supported by prior research which identified night shift nurses have higher mean energy intakes than those who have never worked a night shift^(50)^. Also reported, that suboptimal dietary intakes are more likely amongst night shift workers^(51,52)^. Working night shifts is associated with an increased risk of type 2 diabetes and CVD^(53,54)^. Our findings highlight the diverse requirements amongst staff and the need for the food environment to adapt to reflect a 24-hour workplace. Further exploration of the inequalities in food provision based on shift patterns is required, especially for night shift staff. Findings will aid implementation of new standards for hospitals requiring 24-hour food provision^(18)^.

Furthermore, in several studies,^(36,37,39)^ staff suggested targeting the perceived unhealthy eating culture and increasing managerial and peer support levels, to improve the quality of the hospital food environment. It was believed that engaging the entire hospital community in cultivating healthier eating habits would facilitate change at a greater scale. It has been suggested that overall, dietary interventions for healthcare staff can lead to significant positive outcomes, such as a reduction in weight, BMI and cholesterol, but they require careful planning, adequate resources and strong organisational support to be effective^(55)^. This theory is supported by research on human behaviour showing that dietary habits strongly depend on environmental cues and social norms, and that people’s food choices often align with those made by those closest to them^(56)^. The results from a British study, which investigated the dietary habits of 26 000 hospital workers^(57)^, indicated that the healthfulness of employees’ diets may positively influence the quality of food consumed by their colleagues. Furthermore, research by Phiri *et al.* ^(58)^ and Ross *et al.* ^(59)^ found that nurses can positively affect their colleagues’ dietary habits, through encouraging healthy eating or sharing recipes. Likewise, a systematic review of interventions aiming to improve hospital staff’s health^(55)^ revealed that influential employees play a crucial role in developing and sustaining healthy habits among their co-workers. This suggests that both leadership and staff could play a significant role in creating healthier social norms within hospitals. Other policy measures such as fiscal policies to encourage the purchase and consumption of healthier options, which have been implemented in a variety of settings and have been reported in improving consumers health^(60)^ may also be a strategy to consider.

### Strengths and limitations

Studies included in the current review were observational and used self-reported data, e.g. the staff’s perceptions collected via focus groups and interviews are prone to underestimation or recall bias^(61)^. Moreover, several publications^(33,34,37,38,41)^ used a subjective definition of ‘healthy eating’, which could have contributed towards response bias^(62)^. Two studies^(34,36)^ explored solely the perceived barriers to healthy eating, which could have led to an overemphasis on the negative elements of the hospital food environment and portray an incomplete picture of its quality. Additionally, most of the reviewed studies^(32–34,36–39,41)^ focussed exclusively on nurses’ opinions and experiences, indicating the need for more research exploring the perceptions of other hospital employees.

The main strength of this systematic review is its adherence to the standardised PRISMA-P protocol^(24)^, which helped to ensure the robustness of the process and findings reproducibility. To minimise the risk of bias, the screening, data extraction and quality appraisal processes were double-blinded and performed independently. Moreover, to ensure the comprehensiveness of the search, grey literature scoping search was conducted. However, this review includes only English-language studies, published in the UK, USA or Australia, which limits the representation of the hospital staff’s views from different cultural contexts. Additionally, as it aims to explore subjective opinions, the review contains a high proportion of publications with a high risk of bias, including the *Nursing Standard* articles^(32,35)^ and the Twitter survey^(11)^. The perceptions included in these publications cannot be verified as coming from hospital staff, which affects the strength of their findings.

### Future recommendations

This systematic review has found that most hospital staff appear to be dissatisfied with their workplace food environment and recognise a need for improvement. Since the nutritional needs and challenges of the hospital staff significantly differ from those of patients and visitors^(18)^, engaging hospital workers in developing strategies aimed at improving the food environment standards could help ensure that the challenges associated with irregular working hours, heavy workloads, limited breaks and emotional demands are considered and adequately addressed. As suggested in three publications from the current review^(35,37,41)^, these strategies could potentially focus on subsiding healthy foods, increasing peer and managerial support and providing 24-hour access to nutritious meals. Moreover, this review has identified the need for more robust, interventional studies using objective measurements and a standardised ‘healthy eating’ definition. The results of such research, combined with the staff’s insights, could aid in developing more focused policies and interventions aiming to improve the hospital food environment and staff quality of life. Furthermore, healthier staff are likely to provide better patient care, as they are less prone to illness^(55)^. Less absenteeism will also lead to increased productivity and significant cost savings^(45,63)^.

### Conclusions

In summary, findings from this review show that despite the ongoing governmental efforts to improve the hospital food environment, most hospital staff remain dissatisfied with its quality and highlight the negative influence on their health and well-being. The hospital food environment is not tailored to meet staff needs, suggesting employees’ engagement in developing policies aiming to improve the quality of the hospital food environment may be beneficial. However, to fully understand hospital staff’s perceptions of their workplace food environment and determine the causality between the quality of this environment and employees’ health and well-being, more robust, interventional studies comprising a wide range of professions are needed.

## Appendices

### Appendix 1. Scopus Search Strategy

(TITLE-ABS-KEY (“Healthcare Staff” OR “Hospital staff” OR “Healthcare professional*” OR “Allied healthcare professional*” OR dietitian* OR dietician* OR dentist* OR midwi* OR nurs* OR doctor* OR “Healthcare worker*” OR “Healthcare employee*” OR “NHS staff*” OR “National health service staff” OR “NHS employee*” OR “National Health Service employee*” OR “Healthcare Assistant*” OR “Hospital Employee*” OR colleague*) AND TITLE-ABS-KEY (opinion* OR view* OR thought* OR attitude* OR acceptability OR satisfaction OR preference* OR complaint* OR feedback OR experienc* OR behavio?r OR “Food choice*” OR relationship* OR perspect* OR observation* OR engagement OR sugges

tion* OR perception* OR belief* OR feel*) AND TITLE-ABS-KEY (canteen OR “staff

room” OR “Vending machin*” OR cafe* OR shop* OR newsagent* OR kitchen* OR “Coffee shop*” OR tearoom* OR “Dining room*” OR diner* OR “Food and drink outlet*” OR “Drinks machine*” OR “Coffee machine*” OR “Water fountain*” OR “Cooking facilit*” OR “Food Purchase*” OR “Self service” OR “Eating area” OR “Break room*” OR “Tea station*” OR “Onsite food outlet*” OR “Food provision*” OR “Food Service*” OR “Retail outlet*” OR food OR drink* OR “Automatic food dispenser*” OR “Hospital food environment” OR restaurant* OR “Food availability” OR “Food accessibility” OR diet* OR catering OR “Fast Food”) AND TITLE-ABS-KEY (england OR scotland OR wales OR “Northern Ireland” OR “United Kingdom” OR “Great Britain” OR “USA” OR “United States of America” OR “Australia”) AND TITLE-ABS-KEY (hospital* OR clinic* OR “acute setting*” OR “private hospital*” OR “NHS trust*” OR “national health service trust*” OR “hospital trust*” OR “public hospital*” OR “A&E” OR “Accident and Emergency” OR “Emergency Department” OR “Emergency care setting”)) AND PUBYEAR > 2009 AND PUBYEAR < 2024 AND (LIMIT-TO (AFFILCOUNTRY, “United Kingdom”) OR LIMIT-TO (AFFILCOUNTRY, “Australia”) OR LIMIT-TO (AFFILCOUNTRY, “United States”)) AND (LIMIT-TO (

LANGUAGE, “English”))
